# Prognostic value of pretreatment lymphocyte-to-monocyte ratio in patients with glioma: a meta-analysis

**DOI:** 10.1186/s12916-023-03199-6

**Published:** 2023-12-05

**Authors:** Yan Wang, Chu Xu, Zongxin Zhang

**Affiliations:** 1 Clinical Laboratory, Huzhou Central Hospital, Affiliated Central Hospital of Huzhou University, The Fifth School of Clinical Medicine Zhejiang Chinese Medical University, Huzhou, 313000 Zhejiang China; 2 Department of Neurosurgery, The Third Affiliated Hospital of Chongqing Medical University, Chongqing, 401120 China

**Keywords:** Lymphocyte-to-monocyte ratio, Glioma, Meta-analysis, Evidence-based medicine, Biomarker

## Abstract

**Background:**

Many studies have explored the prognostic role of the lymphocyte-to-monocyte ratio (LMR) in patients with glioma, but the results have been inconsistent. We therefore conducted the current meta-analysis to identify the accurate prognostic effect of LMR in glioma.

**Methods:**

The electronic databases of PubMed, Web of Science, Embase, and Cochrane Library were thoroughly searched from inception to July 25, 2023. The pooled hazard ratios (HRs) and 95% confidence intervals (CIs) were calculated to estimate the prognostic role of LMR for glioma.

**Results:**

A total of 16 studies comprising 3,407 patients were included in this meta-analysis. A low LMR was significantly associated with worse overall survival (OS) (HR = 1.35, 95% CI = 1.13–1.61, *p* = 0.001) in glioma. However, there was no significant correlation between LMR and progression-free survival (PFS) (HR = 1.20, 95% CI = 0.75–1.91, *p* = 0.442) in glioma patients. Subgroup analysis indicated that a low LMR was significantly associated with inferior OS and PFS in glioma when using a cutoff value of ≤ 3.7 or when patients received mixed treatment.

**Conclusions:**

This meta-analysis demonstrated that a low LMR was significantly associated with poor OS in glioma. There was no significant correlation between LMR and PFS in glioma patients. The LMR could be a promising and cost-effective prognostic biomarker in patients with glioma in clinical practice.

## Background

Glioma is the most common primary malignant brain tumor, accounting for approximately 27% of all brain and central nervous system tumor [1, 2]. As gliomas are highly heterogeneous and proliferate invasively, therapeutic approaches can be challenging [3]. According to the latest 2021 World Health Organization (WHO) Central Nervous System (CNS) 5 classification [4, 5], adult-type diffuse gliomas are classified into 3 types: (1) astrocytoma, isocitrate dehydrogenase (IDH) mutant (WHO grades 2–4); (2) oligodendroglioma, IDH mutant and 1p/19q codeleted (WHO grades 2 and 3); and (3) glioblastoma (GBM), IDH wild type (WHO grade 4) [5]. GBM is the most common and aggressive type of primary brain tumor, which comprises up to 50% of all gliomas [6]. The survival outcomes of patients with glioma have not improved over the past several decades, despite treatment options, such as surgery, chemotherapy, and radiotherapy. The prognosis of GBM is poor, with a median overall survival (OS) of 15 months and a 5-year survival rate of only approximately 5% [7, 8]. Therefore, there is an urgent need to identify novel and effective prognostic markers for glioma.

Evidence suggests that the tumor microenvironment, notably the inflammatory response, may promote cancer development and progression and is associated with systemic inflammation [9]. A number of hematological indicators have been reported to be highly predictive of cancer patient prognosis in recent years, such as the neutrophil-to-lymphocyte ratio (NLR) [10], platelet-to-lymphocyte ratio (PLR) [11], systemic immune-inflammation index (SII) [12], and lymphocyte-to-monocyte ratio (LMR) [13]. For example, a review involving 11 studies showed that PLR could be a useful marker to aid in the prognosis of GBM [14]. Another important systematic review indicated that the NLR was a cost-effective and low-cost tool that was associated with tumor grading and overall survival (OS) in patients with glioma [15]. The LMR is derived by dividing the absolute lymphocyte count by the absolute monocyte count. Many studies have reported that LMR is a significant prognostic marker for various solid tumors, including thyroid cancer [16], renal cell carcinoma [17], small cell lung cancer [18], ovarian cancer [19], and cholangiocarcinoma [20]. Previous studies have also explored the prognostic effect of LMR in patients with glioma, but the results were controversial [21–36]. For example, some researchers reported that a low LMR was significantly associated with poor survival in glioma [30, 31, 34, 35]. However, some other clinicians showed that there was no significant correlation between LMR and the prognosis of glioma patients [22–24, 27]. Therefore, we performed a meta-analysis to identify the precise prognostic function of LMR in glioma.

## Methods

### Study guidelines

This meta-analysis was carried out in accordance with the Preferred Reporting Items for Systematic Reviews and Meta-Analyses (PRISMA) guidelines [37].

### Ethics statement

In our study, no human or animal experiments were conducted, and no primary personal information will be gathered. Therefore, no ethical approval or consent was needed.

### Search strategy

The electronic databases of PubMed, Web of Science, Embase, and Cochrane Library were thoroughly searched from inception to July 25, 2023. The detailed search strategy was as follows: (LMR or lymphocyte-to-monocyte ratio or lymphocyte-monocyte ratio or lymphocyte-to-monocyte ratio) and (glioma or glioblastoma or glial tumor or astrocytoma or oligodendroglioma). Only publications in English were considered. Furthermore, references cited in these studies were also reviewed to identify additional published articles not indexed in the standard databases.

### Eligibility criteria

The inclusion criteria were as follows: (1) the diagnosis of glioma was pathologically or histologically confirmed; (2) the association between LMR and survival outcomes in glioma was investigated; (3) the hazard ratios (HRs) and 95% confidence intervals (CIs) for survival outcomes were reported or could be calculated by given information; (4) a cutoff value to define low and high LMR was identified; and (5) the study was published in the English language. The exclusion criteria were as follows: (1) case reports, reviews, letters, conference abstracts, and comments; (2) animal studies; and (3) studies with overlapping patients.

### Data extraction

Two investigators (Y.W. and C.X.) independently reviewed the eligible studies and extracted data from the included studies. All disagreements were resolved by discussion until consensus. The following information was extracted: first author’s name, year of publication, country, study period, sample size, age, sex, WHO grade, histology, treatment, cutoff value, methods to determine cutoff value, follow-up, survival outcomes, survival analysis type, and HRs with 95% CIs. The primary survival endpoint was OS, and the secondary survival endpoint was progression-free survival (PFS).

### Quality assessment

The Newcastle‒Ottawa Scale (NOS) was used to evaluate the quality of each selected study by two independent reviewers (C.X. and Z.Z.) [38]. The NOS assesses the quality of studies in the following aspects: selection (4 points), comparability (2 points), and results and adequacy of follow-up (3 points). The NOS score ranges from 0 to 9, and studies with NOS scores ≥ 6 are considered high-quality.

### Statistical analysis

The pooled HRs and 95% CIs were calculated to estimate the prognostic role of LMR for glioma patients. The heterogeneity among studies was evaluated by using Cochran’s Q test and Higgins *I*^2^ statistic. A random-effects model was applied when significant heterogeneity was observed, as measured by an I^2^ greater than 50% or a P value less than 0.1. Otherwise, a fixed-effects model was adopted. Subgroup analysis stratified by diverse factors and meta-regression analysis were conducted to explore the source of heterogeneity. Sensitivity analysis was conducted to evaluate the stability of the combined results. Begg’s test and Egger’s test were used to assess publication bias. Stata software version 12.0 was used to conduct all statistical analyses (Stata Corporation, College Station, TX, USA). *P* values < 0.05 were defined as statistically significant.

## Results

### Search results

**Fig. 1:**
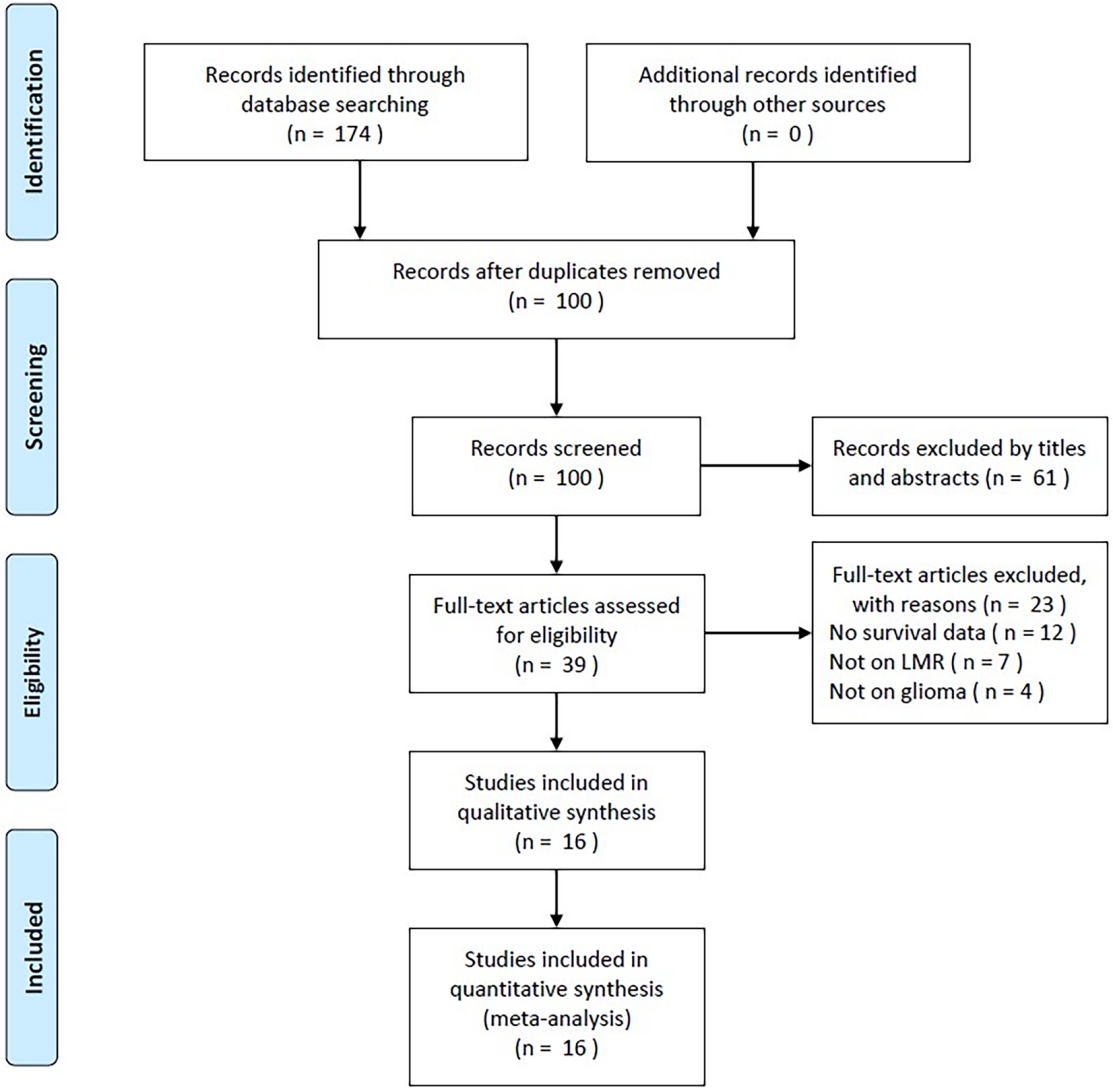
PRISMA flowchart of literature search and study selection

As shown in Fig. 1, the initial literature search identified a total of 174 records, and 100 studies remained after the removal of duplicates. Through screening titles and abstracts, 61 studies were further discarded because they were irrelevant studies or animal experiments. Subsequently, the remaining 39 studies were examined by full-text reading. Then, 23 studies were eliminated for the following reasons: no survival data provided (*n* = 12), not concerning LMR (*n* = 7), and not concerning glioma (*n* = 4). Ultimately, a total of 16 studies comprising 3407 patients [21–36] were included in this meta-analysis (Fig. 1).

### Characteristics of included studies

The baseline characteristics of the included studies are shown in Table 1. They were published from 2016 to 2023. The sample sizes ranged from 22 to 592, and the median value was 193. Thirteen studies were performed in China [21–25, 27, 29–33, 35, 36], and one each in Australia [26], India [28], and Bulgaria [34]. Ten studies recruited patients with glioma [23–27, 29–32, 35], and six studies enrolled patients with GBM [21, 22, 28, 33, 34, 36]. The cutoff values of LMR ranged from 1.87 to 5, with a median value of 3.7. Twelve studies used the receiver operating characteristic (ROC) curve to determine the cutoff value [23, 24, 26, 27, 29–36], and four studies adopted X-tile software [21, 22, 25, 28]. Fifteen studies included reporting on the prognostic role of LMR for OS [21–27, 29–36], and three studies presented the association between LMR and PFS [28, 29, 33]. Fourteen studies derived HRs and 95% CIs by using univariate analysis [21, 22, 24–34, 36], and two studies applied multivariate analysis [23, 35]. The NOS scores of the included studies ranged from 7 to 9, with a median value of 8, which suggested the high quality of eligible studies (Table 1).

**Table 1:** Basic characteristics of included studies in this meta-analysis

Author	Year	Country	Sample size	Study period	Age (year) Median (range)	Gender (M/F)	Tumor grade	Histological type	Treatment	Cut-off value	Cut-off determination	Follow-up (month) Median (range)	Survival outcome	Survival analysis	NOS score
Zhou, X. W	2016	China	84	2013–2014	53 (43–62)	50/34	IV	GBM	Surgery	4.37	X-tile	1–40	OS	Univariate	8
Wang, P. F	2017	China	166	2009–2014	52 (18–80)	96/70	IV	GBM	Surgery	3.7	X-tile	1–45	OS	Univariate	7
Bao, Y	2018	China	219	2012–2017	≥ 50 years: 146 < 50 years: 73	124/95	I–IV	Glioma	Surgery	3.7	ROC curve	1–60	OS	Multivariate	8
He, Z. Q	2019	China	154	2001–2016	40	92/62	III	Glioma	Mixed	4.33	ROC curve	1–160	OS	Univariate	9
Zhang, Z. Y	2019	China	592	2011–2016	42	335/257	II–IV	Glioma	Surgery	2.94	X-tile	32	OS	Univariate	8
Chim, S. T	2021	Australia	64	1989–2018	51.5	44/20	II–IV	Glioma	Mixed	2.86	ROC curve	1–275	OS	Univariate	7
He, Q	2021	China	105	2013–2019	50 (18–79)	57/48	III–IV	Glioma	Surgery	5.0	ROC curve	1–80	OS	Univariate	8
Madhugiri, V. S	2021	India	408	2007–2017	55	280/128	IV	GBM	Surgery	1.87	X-tile	1–113	PFS	Univariate	8
Xie, T	2021	China	318	2001–2014	44 (5–78)	194/124	III–IV	Glioma	Mixed	3.86	ROC curve	1–180	OS, PFS	Univariate	9
Yan, P	2021	China	162	2012–2018	45 (7–82)	88/74	II–IV	Glioma	Mixed	4.26	ROC curve	1–96	OS	Univariate	7
Chen, X. Y	2022	China	199	2015–2020	< 60 years: 143 ≥ 60 years: 56	111/88	III–IV	Glioma	Mixed	4.47	ROC curve	1–30	OS	Univariate	8
Qi, Z	2022	China	214	2001–2013	41 (5–79)	131/83	II–III	Glioma	Mixed	4.81	ROC curve	1–144	OS	Univariate	7
Shi, X	2022	China	232	2014–2018	< 65 years: 193 ≥ 65 years: 39	127/105	IV	GBM	Mixed	2.78	ROC curve	1–70	OS, PFS	Univariate	8
Stoyanov, G. S	2022	Bulgaria	22	2018–2021	66 (50–86)	15/7	IV	GBM	Mixed	2.22	ROC curve	8 (1–26)	OS	Univariate	8
Yang, C	2022	China	187	2016–2019	50 (21–81)	111/76	II–IV	Glioma	Mixed	2.3	ROC curve	1–50	OS	Multivariate	7
Duan, X	2023	China	281	2015–2018	≤ 65 years: 223 > 65 years: 58	155/126	IV	GBM	Mixed	3.57	ROC curve	19 (3.5–63)	OS	Univariate	8

*GBM* glioblastoma, *M* male, *F* female, *ROC*, receiver operating characteristic, *OS* overall survival, *PFS* progression-free survival, *NOS* Newcastle–Ottawa Scale

### LMR and OS

**Fig. 2:**
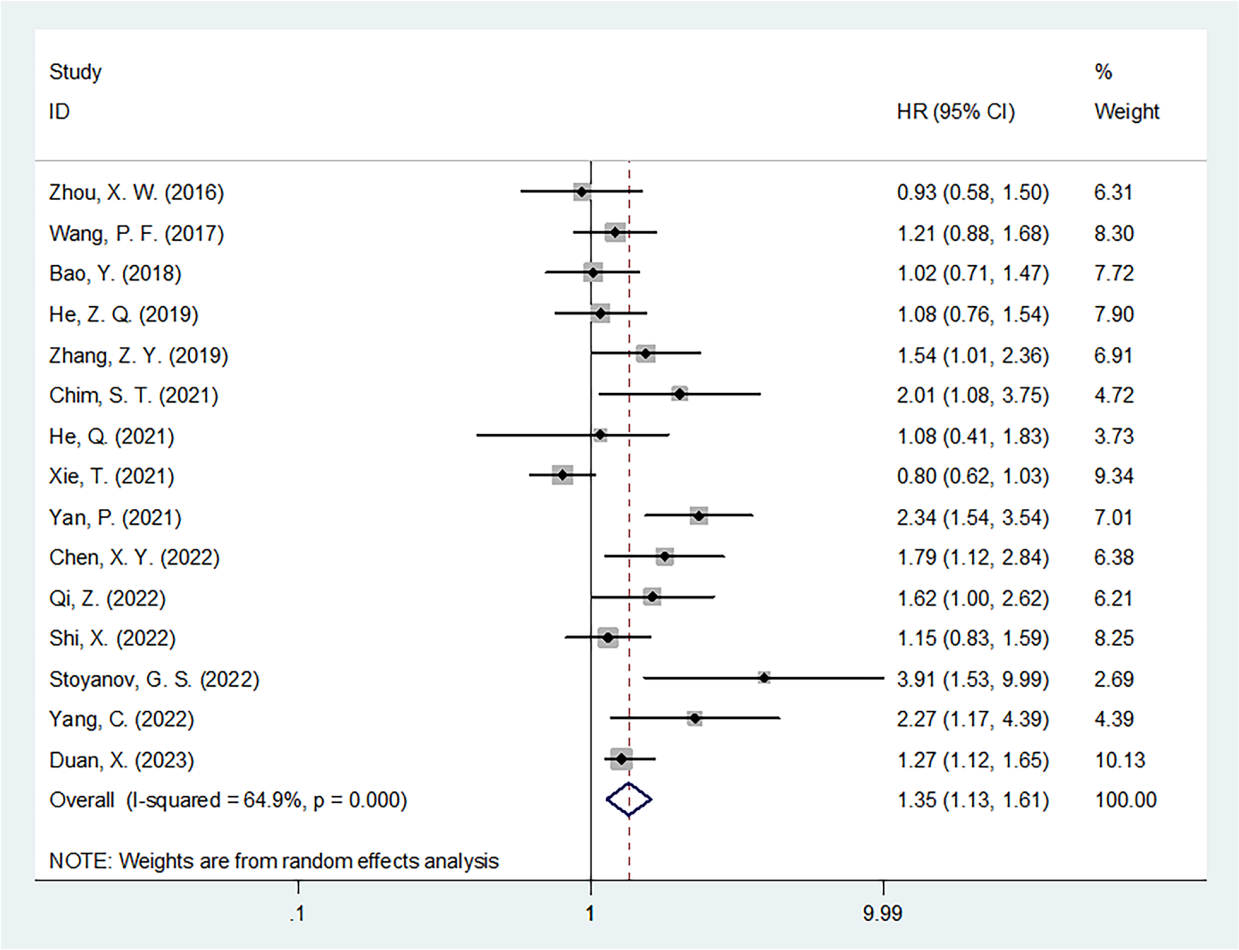
Forest plot of association between LMR and OS in patients with glioma

A total of 15 studies with 2999 patients [21–27, 29–36] provided the prognostic value of LMR for OS in glioma. As the heterogeneity was significant (*I*^2^ = 64.9%, *p* < 0.001), a random-effects model was used. As shown in Table 2 and Fig. 2, the pooled results were HR = 1.35, 95% CI = 1.13–1.61, *p* = 0.001, indicating that a low LMR was significantly correlated with poor OS in patients with glioma. Subgroup analysis showed that the prognostic effect of LMR for OS was not influenced by country or histology (all *p* < 0.05; Table 2). Moreover, low LMR remained a significant prognostic indicator for poor OS in the following subgroups: sample size < 200 (*p* = 0.001), mixed treatment (*p* = 0.002), cutoff value of ≤ 3.7 (*p* < 0.001), cutoff determination by ROC curve (*p* = 0.002), and univariate analysis (*p* = 0.002) (Table 2). Meta-regression analysis showed that country, sample size, histological type, treatment, cutoff value, cutoff determination, and survival analysis were not the only factors that contributed to the significant heterogeneity (all *p* > 0.05; Table 2). The significant heterogeneity could be the result of multiple factors working together.

**Table 2:** Subgroup analysis of prognostic role of LMR for overall survival in patients with glioma

Subgroups	No. of studies	No. of patients	Effects model	HR (95%CI)	*p*	Heterogeneity		Mete-regression *p*
						*I*^2^ (%)	Ph	
Total	15	2999	Random	1.35 (1.13–1.61)	0.001	64.9	< 0.001	
Country								0.059
China	13	2913	Random	1.28 (1.08–1.51)	0.005	62.1	0.001	
Others	2	86	Fixed	2.46 (1.47–4.14)	0.001	25.4	0.247	
Sample size								0.156
< 200	9	1143	Random	1.56 (1.19–2.04)	0.001	61.4	0.008	
≥ 200	6	1856	Random	1.16 (0.94–1.43)	0.166	62.0	0.022	
Histological type								0.646
Glioma	10	2214	Random	1.42 (1.09–1.85)	0.009	72.2	< 0.001	
GBM	5	785	Fixed	1.23 (1.07–1.42)	0.003	46.2	0.114	
Treatment								0.241
Surgery	5	1166	Fixed	1.16 (0.96–1.39)	0.124	0	0.535	
Mixed	10	1833	Random	1.49 (1.17–1.91)	0.002	75.0	< 0.001	
Cut-off value								0.536
≤ 3.7	8	1763	Fixed	1.31 (1.15–1.48)	< 0.001	46.2	0.072	
> 3.7	7	1236	Random	1.28 (0.92–1.77)	0.140	76.4	< 0.001	
Cut-off determination								0.559
X-tile	3	842	Fixed	1.22 (0.98–1.53)	0.081	16.8	0.301	
ROC curve	12	2157	Random	1.40 (1.13–1.74)	0.002	70.6	< 0.001	
Survival analysis								0.960
Univariate	13	2593	Random	1.35 (1.12–1.63)	0.002	66.2	< 0.001	
Multivariate	2	406	Random	1.45 (0.66–3.15)	0.352	76.9	0.037	

*GBM* glioblastoma, *ROC* receiver operating characteristic

### LMR and PFS

**Fig. 3:**
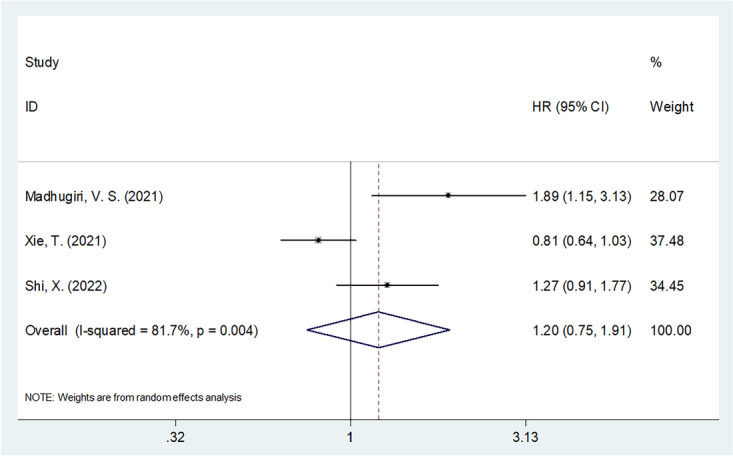
Forest plot of association between LMR and PFS in patients with glioma

Three studies consisting of 958 patients [28, 29, 33] included reporting on the relationship between LMR and PFS in patients with glioma. The random-effects model was applied due to significant heterogeneity (*I*^2^ = 81.7%, *p* = 0.004). The combined data showed that there was a nonsignificant association between LMR and PFS in glioma (HR = 1.20, 95% CI = 0.75–1.91, *p* = 0.442; Table 3 and Fig. 3). Subgroup analysis demonstrated that low LMR was a significant prognostic factor for inferior PFS in the following subgroups: studies in non-China countries (*p* = 0.013), histology of GBM (*p* = 0.011), surgery treatment (*p* = 0.013), cutoff value of ≤ 3.7 (*p* = 0.011), and cutoff value determination of X-tile software (*p* = 0.013) (Table 3).

**Table 3:** Subgroup analysis of prognostic role of LMR for progression-free survival in patients with glioma

Subgroups	No. of studies	No. of patients	Effects model	HR (95%CI)	*p*	Heterogeneity	
						*I*^2^ (%)	Ph
Total	3	958	Random	1.20 (0.75–1.91)	0.442	81.7	0.004
Country							
China	2	550	Random	1.00 (0.65–1.55)	0.997	78.2	0.032
Others	1	408	-	1.89 (1.14–3.11)	0.013	-	-
Histological type							
Glioma	1	318	-	0.81 (0.64–1.03)	0.089	-	-
GBM	2	640	Fixed	1.43 (1.09–1.88)	0.011	40.9	0.194
Treatment							
Surgery	1	408	-	1.89 (1.14–3.11)	0.013	-	-
Mixed	2	550	Random	1.00 (0.65–1.55)	0.997	78.2	0.032
Cut-off value							
≤ 3.7	2	640	Fixed	1.43 (1.09–1.88)	0.011	40.9	0.194
> 3.7	1	318	-	0.81 (0.64–1.03)	0.089	-	-
Cut-off determination							
X-tile	1	408	-	1.89 (1.14–3.11)	0.013	-	-
ROC curve	2	550	Random	1.00 (0.65–1.55)	0.997	78.2	0.032

*GBM* glioblastoma, *ROC* receiver operating characteristic

### Sensitivity analysis

**Fig. 4:**
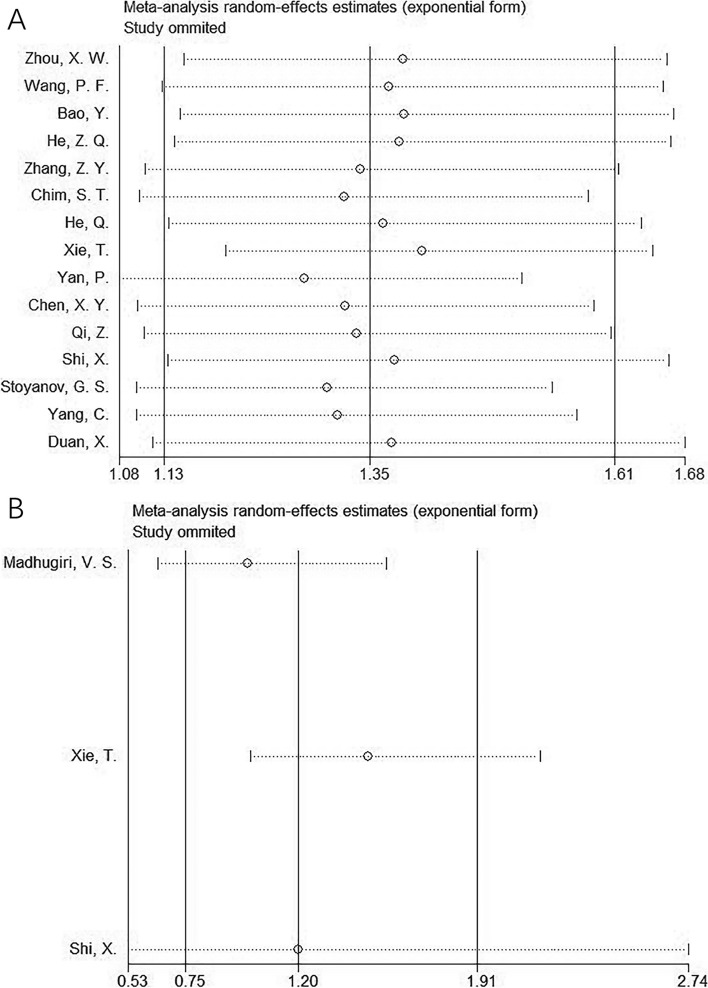
Sensitivity analysis. **A** OS and **B** PFS

Using sensitivity analysis, it was shown that the results of the current meta-analysis were stable and reliable (Fig. 4). OS and PFS results were not significantly affected by any one of the included studies.

### Publication bias

**Fig. 5:**
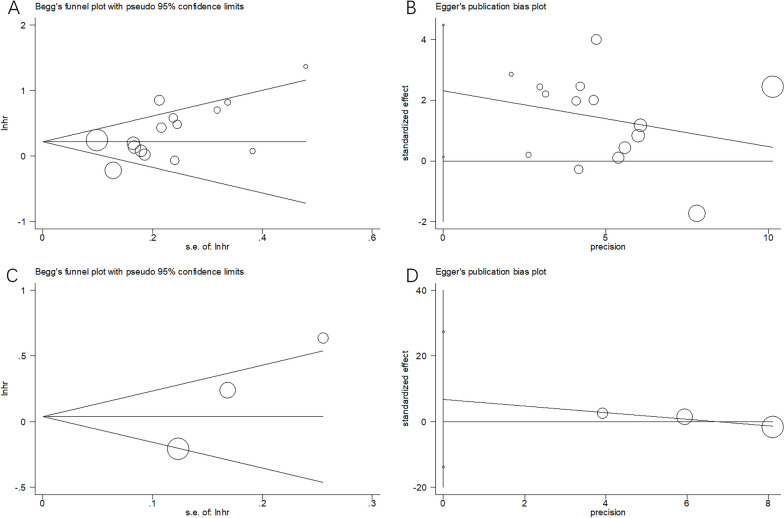
Publication bias test for OS and PFS. **A** Begg’s test for OS, *p* = 0.092; **B** Egger’s test for OS, *p* = 0.150; **C** Begg’s test for PFS, *p* = 0.296; and **D** Egger’s test for PFS, *p* = 0.161

Potential publication bias was tested by using Begg’s test and Egger’s test. As shown in Fig. 5, the shapes of the funnel plots were symmetric. The results were as follows: for OS—Begg’s test, *p* = 0.092, Egger’s test, *p* = 0.150; for PFS—Begg’s test, *p* = 0.296, Egger’s test, *p* = 0.161. These results revealed that there was no significant publication bias in this meta-analysis.

## Discussion

The LMR is calculated by dividing the absolute lymphocyte count by the absolute monocyte count. Therefore, a low LMR could be attributed to low lymphocyte counts and/or high monocyte counts. Although the precise mechanisms of the association between LMR and survival in glioma are not fully elucidated, they can be explained in the following aspects. First, lymphocytes play an important role in cellular antitumor responses. Lymphocytes facilitate the activation of the host immune response to cancer by inhibiting the growth and proliferation of cancer cells through direct cytotoxic cell death in immune surveillance [39]. Lymphocytopenia might be related to an inappropriate immune response during tumor growth [40]. Deficiencies in peripheral lymphocytes may result in tumor cell proliferation and metastasis when antitumor responses are impaired [39]. Moreover, cytokines impair T-lymphocytic function and cell-mediated immunity when pro-inflammatory status is present [41]. In contrast, monocytes can differentiate into tumor-associated macrophages (TAMs) and dendritic cells to promote tumorigenesis and suppress the immune response in the tumor microenvironment (TME) [42]. Angiogenesis may be promoted by TAMs, which produce growth factors and chemokines that contribute to malignant progression [43]. Moreover, in the TME, monocytes from the peripheral blood enter tumor sites constantly and release soluble inhibitory factors and inhibitory molecules that inhibit the immune system’s defenses against tumors [43, 44].

The prognostic value of LMR in patients with glioma was inconsistent according to previous studies. In the current meta-analysis, we retrieved the literature and synthesized the data from 16 studies with 3407 cases. Our meta-analysis indicated that a low LMR was a significant prognostic marker for poor OS in glioma. However, there was a nonsignificant correlation between LMR and PFS. Furthermore, a low LMR was significantly associated with inferior OS and PFS in glioma when using a cutoff value of ≤ 3.7 or when patients received mixed treatment. Sensitivity analysis and publication bias tests confirmed the reliability of our results. Taken together, this meta-analysis demonstrated that a low LMR was a significant prognostic biomarker for long-term survival in patients with glioma. To our knowledge, this is the first meta-analysis investigating the prognostic importance of LMR in glioma patients.

In recent years, many meta-analyses have also reported the prognostic role of LMR in various cancer types [45–50]. Hamid et al. showed that a low LMR was associated with poorer OS and disease-free survival (DFS) in rectal cancer in a meta-analysis with 6683 patients [45]. Gao and colleagues revealed that a low LMR was associated with poor OS and reduced DFS/PFS in nasopharyngeal carcinoma through a meta-analysis involving 3773 patients [46]. Dotto-Vasquez et al. performed a meta-analysis including 19 studies and indicated that cholangiocarcinoma patients with low values of LMR were associated with worse OS and poor time to recurrence (TTR) [47]. In a recent meta-analysis comprising 8361 cases, it was reported that decreased pretreatment LMR was significantly correlated with reduced PFS and worse OS in lung cancer [48]. Another large-scale meta-analysis with 10,446 patients found that a low LMR was associated with inferior OS and PFS in lymphoma [49]. Cai and colleagues showed that a lower LMR was associated with poorer OS and PFS in ovarian cancer in their meta-analysis enrolling 2809 patients [50]. In the current meta-analysis, we identified the significant prognostic effect of LMR for OS in glioma, which was in line with findings in other solid tumors.

Notably, this meta-analysis showed that there was a nonsignificant correlation between LMR and PFS in patients with glioma (HR = 1.20, 95% CI = 0.75–1.91, *p* = 0.442). The negative results could be due to the following reasons. First, the sample size in the LMR and PFS analyses was small. Only three studies with 958 patients were included for analysis. Second, the survival duration for GBM patients was relatively short, with a median survival of 15 months [51]. Moreover, the median PFS after recurrence was only.

1.8 months in glioma patients [52]. Therefore, the follow-up in PFS was not long, so the prognostic role of LMR is nonsignificant. Third, the heterogeneity was significant, which could be a potential reason for this negative result.

The present meta-analysis has some limitations. First, all included studies were retrospective, and most of them were conducted in Asian countries. Therefore, selection bias may be introduced. Second, significant heterogeneity among studies was detected for the analysis of OS and PFS. We adopted a random-effects model or fixed-effects model according to the level of heterogeneity. Third, the cutoff values of LMR were not uniform in the included studies. Our meta-analysis showed that LMR ≤ 3.7 could be an optimal cutoff value for prognostication in glioma. A standard cutoff value of LMR in glioma prognosis needs to be established and validated in future studies. Therefore, due to several limitations, multicenter prospective trials are still needed to verify the results of our meta-analysis. Therefore, due to several limitations, multicenter prospective trials are still needed to verify the results of our meta-analysis.

## Conclusions

In summary, this meta-analysis demonstrated that a low LMR was significantly associated with poor OS in glioma. LMR could be a promising and cost-effective prognostic biomarker in patients with glioma in clinical practice.

## Data Availability

The data that support the findings of this study are available from the corresponding author upon reasonable request.

## Abbreviations

CI: Confidence interval
DFS: Disease-free survival
GBM: Glioblastoma
HR: Hazard ratio
LMR: Lymphocyte-to-monocyte ratio
NLR: Neutrophil-to-lymphocyte ratio
NOS: Newcastle-Ottawa Scale
OS: Overall survival
PFS: Progression-free survival
PLR: Platelet-to-lymphocyte ratio
PRISMA: Preferred Reporting Items for Systematic Reviews and Meta-Analyses
SII: Systemic immune-inflammation index
TAMs: Tumor-associated macrophages
TME: Tumor microenvironment
TTR: Time to recurrence
WHO: World Health Organization
